# The direct and sustained consequences of severe placental hypoxia on vascular contractility

**DOI:** 10.1371/journal.pone.0202648

**Published:** 2018-08-24

**Authors:** Philippe Vangrieken, Salwan Al-Nasiry, Ger M. J. Janssen, Antje R. Weseler, Marc E. Spaanderman, Aalt Bast, Paul M. H. Schiffers

**Affiliations:** 1 Department of Pharmacology and Toxicology, Faculty of Health, Medicine and Life Sciences, Maastricht University, Maastricht, The Netherlands; 2 Department of Obstetrics and Gynecology, Maastricht University Medical Centre, Maastricht, The Netherlands; University of Southampton, UNITED KINGDOM

## Abstract

**Introduction:**

Preeclampsia is a major health problem in human pregnancy, severely complicating 5–8% of all pregnancies. The emerging molecular mechanism is that conditions like hypoxic stress trigger the release of placental messengers into the maternal circulation, which causes preeclampsia. Our objective was to develop an *in vitro* model, which can be used to further elucidate the molecular mechanisms of preeclampsia and which might be used to find a remedy.

**Methods:**

Human non-complicated term placentas were collected. Placental explants were subjected to severe hypoxia and the conditioned media were added to chorionic arteries that were mounted into a myograph. Contractile responses of the conditioned media were determined, as well as effects on thromboxane-A_2_ (U46619) induced contractility. To identify the vasoactive compounds present in the conditioned media, specific receptor antagonists were evaluated.

**Results:**

Factors released by placental explants generated under severe hypoxia induced an increased vasoconstriction and vascular contractility to thromboxane-A_2_. It was found that agonists for the angiotensin-I and endothelin-1 receptor released by placental tissue under severe hypoxia provoke vasoconstriction. The dietary antioxidant quercetin could partially prevent the acute and sustained vascular effects in a concentration-dependent manner.

**Discussion:**

Both the acute vasoconstriction, as well as the increased contractility to U46619 are in line with the clinical vascular complications observed in preeclampsia. Data obtained with quercetin supports that our model opens avenues for e.g. nutritional interventions aimed at treating or preventing preeclampsia.

## Introduction

Preeclampsia (PE) is the most frequently encountered severe complication during pregnancy with a clinical spectrum ranging from relatively mild to life-threatening [1, 2]. It is not simply *de novo* onset of hypertension and proteinuria, but rather a syndrome involving multiple organs, which results in end-organ dysfunction [3].

The etiology of PE is enigmatic and except for delivery no treatment exists, which is associated with iatrogenic prematurity and lifelong disabilities for mother and child [4, 5]. Prevention and solid prediction are still not possible, and symptomatic clinical management is mainly directed at preventing maternal morbidity and mortality [3].

The leading hypothesis considers disturbed placental development during the first trimester of pregnancy to be the main cause [3]. Impaired remodelling of maternal spiral arteries is considered to be crucial in the onset of PE. During PE there is an impaired invasion of trophoblast cells into the inner third of the myometrium [6, 7]. As a consequence, no trophoblastic plugs are formed in the ends of the spiral arteries, which is associated with early perfusion of the placenta in the first trimester of pregnancy and impaired remodelling of the spiral arteries in the second and third trimester [8, 9]. Consequently, maternal blood enters the intervillous space at greater velocity creating relatively high fluctuations in placental perfusion and subsequently to placental hypoxia and reoxygenation [8, 10].

Evidence is accumulating, that oxidative stress by excessive production of reactive oxygen species (ROS) in PE increases trophoblast turnover, resulting in an accelerated differentiation of trophoblast cells followed by the release of specific placenta-secreted messengers (PSMs) into the maternal circulation that induce endothelial dysfunction, vasoconstriction, increased blood coagulation and inflammation [11–16]. The exact nature of the PSMs and their specific interaction with maternal cells is still enigmatic.

As Goulopoulou & Davidge et al. discussed, PE is unlikely the result of a single factor/molecular pathway. It involves various circulating factors acting on the maternal vascular wall that disturb the balance between vasodilatory and vasoconstrictor mechanisms [17]. The diversity in the nature of potential instigators and their sources in PE, as well as the complex interactions with maternal targets, underlines the need for further investigation of the common converging pathways that link placental dysfunction with systemic maternal vascular pathology [17].

Redman et al. showed that PE is not only an endothelial disease but also a disorder where the inflammatory changes of a normal pregnancy are exaggerated. These changes involve the endothelium, but also other components of the inflammatory network, including inflammatory leukocytes. This resulting systemic inflammation is not only associated with changes of the acute phase response like fever, increased CRP and angiotensinogen levels, but also with metabolic responses such as insulin resistance [16, 18]. Furthermore, a list of bioactive circulating factors released by syncytiotrophoblasts, which are altered in PE was presented [16]. This list includes corticotrophin-releasing hormone (CRH) that is known to have both peripheral and central actions. Its peripheral actions are mediated by the CRH-R1/2 receptors, both expressed on the endothelium. The CRH-R2 is known to stimulate endothelium release of endothelin-1 (ET-1), a pro-inflammatory factor, which is also known to be involved in the genesis of preeclamptic hypertension [16, 19]. Besides changes in the ET-1 signaling pathways, alterations in vascular angiotensin signaling pathways have been observed in many models of many vascular beds and have previously been linked to preeclamptic hypertension [5].

The major ethical barrier for studying the pathophysiology of PE and testing therapeutic interventions in pregnant woman underline the need for a human *in vitro* model for PE [20, 21].

This prompted us to develop an *in vitro* model for PE, which was used to subject placental explants to severe hypoxia to mimic the stress condition in PE and to collect PSMs. Furthermore, the acute and sustained vascular functional potencies of PSMs generated under severe hypoxia were determined on chorionic arteries since these arteries do not have an autonomic nerve supply and depend on the local release of vasoactive compounds, which in fact permits investigation of direct actions of PSMs. To identify the vasoactive compounds in PSMs, specific receptor antagonists were tested. The food derived antioxidant quercetin was tested in our *in vitro* model of PE to determine whether a dietary intervention could prevent a disturbed release of PSMs in response to hypoxic stress [22].

## Methods

### Patient characteristics

Human term placentas (>38 weeks gestational age) were collected from normal vaginal or caesarean deliveries performed by the Department of Obstetrics, Gynaecology at the Academic Hospital of Maastricht (azM). For this study, a total of 52 placentas were used within one hour after delivery. All experiments were approved by the Medical Ethics Committee Academic Hospital Maastricht and Maastricht University (METC 16-4-047). A total of 52 women were included in the study.

### Chemicals

Acetylcholine (ACh), bradykinin (BK), sodium nitroprusside (SNP), carbachol (Car), angiotensin II (AngII), endothelin 1 (ET-1), serotonin (5-HT, 5-hydroxytriptamine), losartan (Los), bosentan (Bos) and ketanserin (Ket) (Sigma Chemical Co., St Louis, MO, USA). U46619, a thromboxane-A_2_ (TXA_2_) agonist analogue (Enzo Life sciences, USA). HEPES buffer containing (in mM): NaCl 143.3, KCl 4.7, MgSO_4_ 1.2, KH_2_PO_4_ 1.2, CaCl_2_ 2.5, glucose 5.5 and HEPES 15 (pH = 7.4). Krebs Ringer bicarbonate buffer (KRB) containing (in mM): NaCl 118.3, KCl 4.7, CaCl_2_ 2.5, MgSO_4_ 1.2, KH_2_PO_4_ 1.2, NaHCO_3_ 25.0 and glucose 11.1 (pH = 7.4).

### Villous explant isolation and PSMs collection

**Fig 1:**
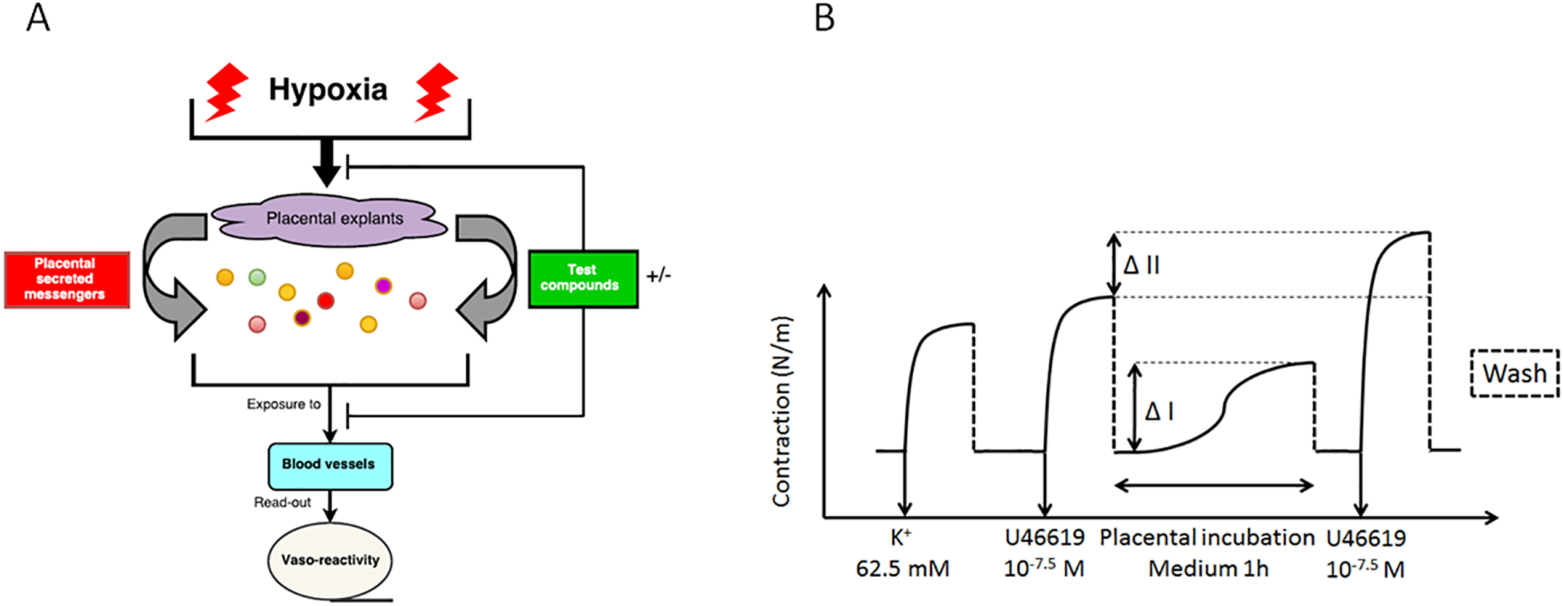
*In vitro* model for severe placental hypoxia and testing the vascular effect of PSMs. Schematic overview of the *In vitro* model for PE where placental explants are exposed to severe hypoxia and PSMs are tested on chorionic arteries. Differences in contractile response are used as read-out (A) and a schematic overview for measuring the acute (ΔI) and sustained (ΔII) vascular effect of PSMs (B).

Three placentas from non-complicated caesarean deliveries were processed directly after delivery. Specimens were collected from the central region of the placentas at the maternal side. The basal plate of the specimens was removed and the remaining tissue was rinsed in an HEPES solution. Two times 30 g minced tissue of each placenta was transferred into 200 ml pre-warmed (37°C) HEPES buffer. This ratio was based on reference values for total maternal blood volume and placental weight [23, 24]. Subsequently, 6 bottles were placed in a water bath at 37°C for 3 hours where 3 bottles were constantly aerated with room air (21% O_2_) as standard culture condition and three bottles with 100% N_2_ to expose the placental cells to severe hypoxia. Based on the findings of Butler et al, after 40 min aeration with 100% N_2_ a steady state concentration of 3% (0.25 ppm) of the original dissolved O_2_ was reached. [25] (Fig 1A). After these exposures, the solutions were centrifuged at 500 *g* for 10 minutes at 4°C to remove cell debris while retaining particles such as micro-vesicles and exosomes, size range 100–1000 and <100 nm respectively [26]. The resulting conditioned medium was stored in aliquots of 3 ml at -80 °C until use.

### Chorionic artery isolation and mounting into a myograph

Placental areas from the chorion plate containing chorionic arteries were macroscopically selected and excised from both vaginal and caesarean deliveries. The specimen was immediately immersed in HEPES solution (4°C) and pinned in a Sylgaard dish with the foetal side orientated upwards. Second and third order branches of the chorionic arteries were dissected. Arterial segments of 2 mm were mounted in a myograph for recording of isometric tension development (DMT, Aarhus, Denmark). The organ chamber was filled with HEPES-buffer that was maintained at 37°C. All arterial preparations were stretched to the diameter at which their individual mechanical performance was maximal. To achieve this, the arterial diameter was stepwise increased and the preparations were intermittently exposed to 65.5 mM potassium-HEPES until maximal contractile responses were obtained. The value of these KCl-contractions was presented as K^+^65.5 mM. To examine all test conditions per placenta, a sufficient number of chorionic arterial segments were isolated. In this way biological variation between different placentas was taken into account. The 175 blood vessel segments used for this study had a diameter of 549 ± 61 μm.

### Functional characterization of chorionic arteries

For the characterisation of the chorionic arteries, the following drugs were used: for vasodilatation ACh, BK, SNP and Car and for vasoconstriction: AngII, ET-1, 5-HT and U46619. Drug solutions were prepared daily and protected from light until use. All drugs were dissolved and diluted in miliQ, except for U46619, which was dissolved in absolute ethanol. Relaxation responses were expressed as a percentage of the maximal contraction achieved by either 65.5 mM KCl or 30 nM U46619.

### Tissue viability

Placental tissue used for the PSM collection was evaluated for viability for both the standard culture (21% O_2_) and the severe hypoxic condition by studying lactate dehydrogenase (LDH) activity using cytotoxicity detection kit (Cat. No.11644793001, Roche, Mannheim, Germany). LDH levels, which are indicative of cell death were measured in the conditioned medium from both conditions for all three placentas.

### Vasoactive responses to PSMs

Chorionic arteries were first pre-contracted by U46619 (30 nM). After washing and complete relaxation (±30 minutes), 3 ml of the thawed conditioned media containing PSMs of either the standard culture (21% O_2_) or the severe hypoxic condition, was added to the organ bath containing 4 ml of HEPES buffer. After 1 hour the PSM induced vasocontraction was measured, followed by a washing step (Fig 1BI). After complete relaxation (±30 minutes), the blood vessels were pre-contracted again by U46619 (30 nM). Differences between the vascular contractile response of the 2^nd^ and 1^st^ pre-contraction were calculated (Fig 1BII).

### Characterisation of vasoactive PSMs released under hypoxia

The vascular contractile response induced by conditioned media containing PSMs released under severe hypoxia was tested when the blood vessels were pre-incubated with the following antagonists: 30 μM Los, 30 μM Bos, 30 μM Ket (AT-1, ET-1 and 5-HT_2_-receptor blockers) or 30 μM Bos + Los.

### Effects of quercetin on the release of PSMs under hypoxia

At the start of the PSMs generation procedure, quercetin (0.3, 1, 3, 10 and 20 μM) was added to 200 ml pre-warmed HEPES buffer (37°C) containing 30 g placental tissue. This was again followed by 3 hours 100% N_2_ aeration. Differences in vascular contractile response to these generated PSMs as well as changes in sustained responsiveness to U46619 were tested.

### Data analysis

Results are expressed as mean ± SEM and n refers to the number of placentas from which blood vessels were isolated. Contractile responses are expressed as a percentage of the maximal contractile response to KCl 65.5 mM. By using GraphPad Software inc. La Jolla, CA, USA), the maximal contractile response (E_max_) and the logarithm of the half maximal effective concentration (pEC_50_) of various compounds were calculated. For the viability test, a nonparametric Mann-Whitney test was used. For the analysis of the vascular contractile effect to PSMs, ET-1, or AngII and the characterisation of the placental vessels, a two-way ANOVA followed by a Bonferroni posthoc test and a nonparametric Mann-Whitney test were used. To determine the consistency of the of PSMs and the effect of quercetin on the release of PSMs under hypoxia, a two-way ANOVA followed by a Bonferroni posthoc test and a nonparametric Kruskal-Wallis test followed by a Dunn’s multiple comparison test were used. A p-value < 0.05 was considered as significant difference and presented as follows: Ns: P > 0.05, *P ≤ 0.05, ** P ≤ 0.01, *** P ≤ 0.001.

## Results

### Functional characterisation chorionic arteries

#### Vasodilation

**Fig 2:**
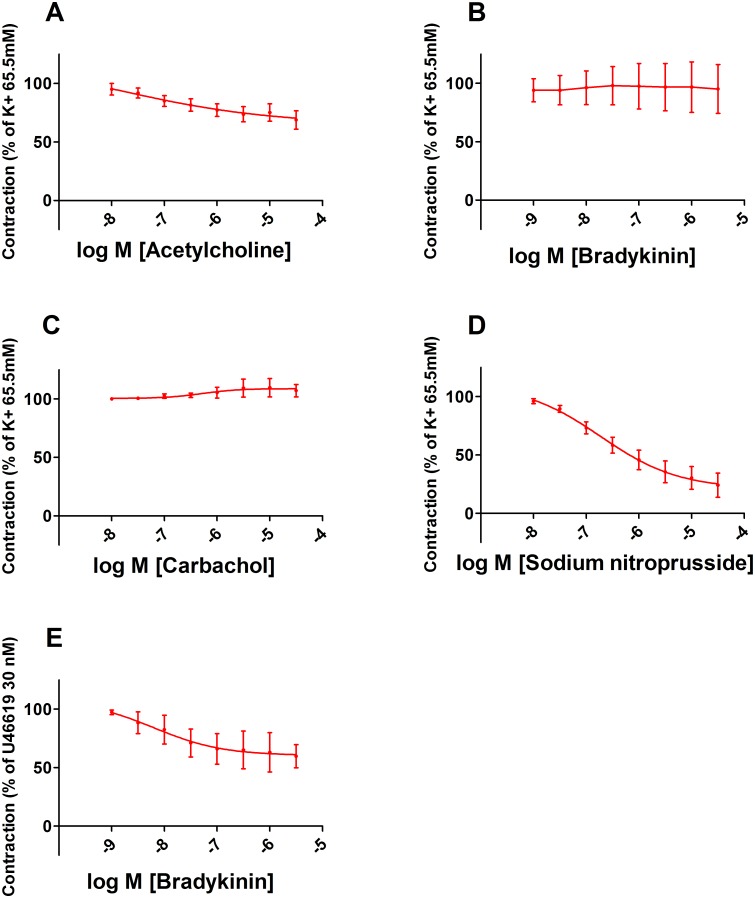
Vasodilation of chorionic arteries. Concentration-dependent relaxation of placental chorionic arteries induced by ACh (n = 6) (A), BK (n = 3) (B), Car (n = 3) (C) or SNP (n = 5) (D), after a pre-contraction induced by KCl 65.5 mM, except for BK, which was after a pre-contraction by U46619 (n = 4) (E).

The NO donor sodium nitroprusside (SNP, 30 μM) induced vasorelaxation up to 76 ± 23%, indicating that chorionic arteries are NO-sensitive. No clear vascular relaxation was induced by ACh, BK or Car after pre-contraction (65.5 mM KCl). The results indicate that ACh, BK or Car are not able to activate eNOS to induce a relevant NO-dependent relaxation. However, after contraction by U46619 (30 nM), BK (3 μM) induced vasodilation up to 40 ± 20% suggesting that endothelium-derived hyperpolarizing factor (EDHF) which is released in response to BK initiates vasorelaxation. A pEC_50_ could be calculated from the concentration-dependent relaxation curves for ACh (7.9 ± 0.8), SNP (6.7 ± 0.5) and BK (7.2 ± 1.1) pre-contracted by U46619 (30 nM) (Fig 2).

#### Vasoconstriction

**Fig 3:**
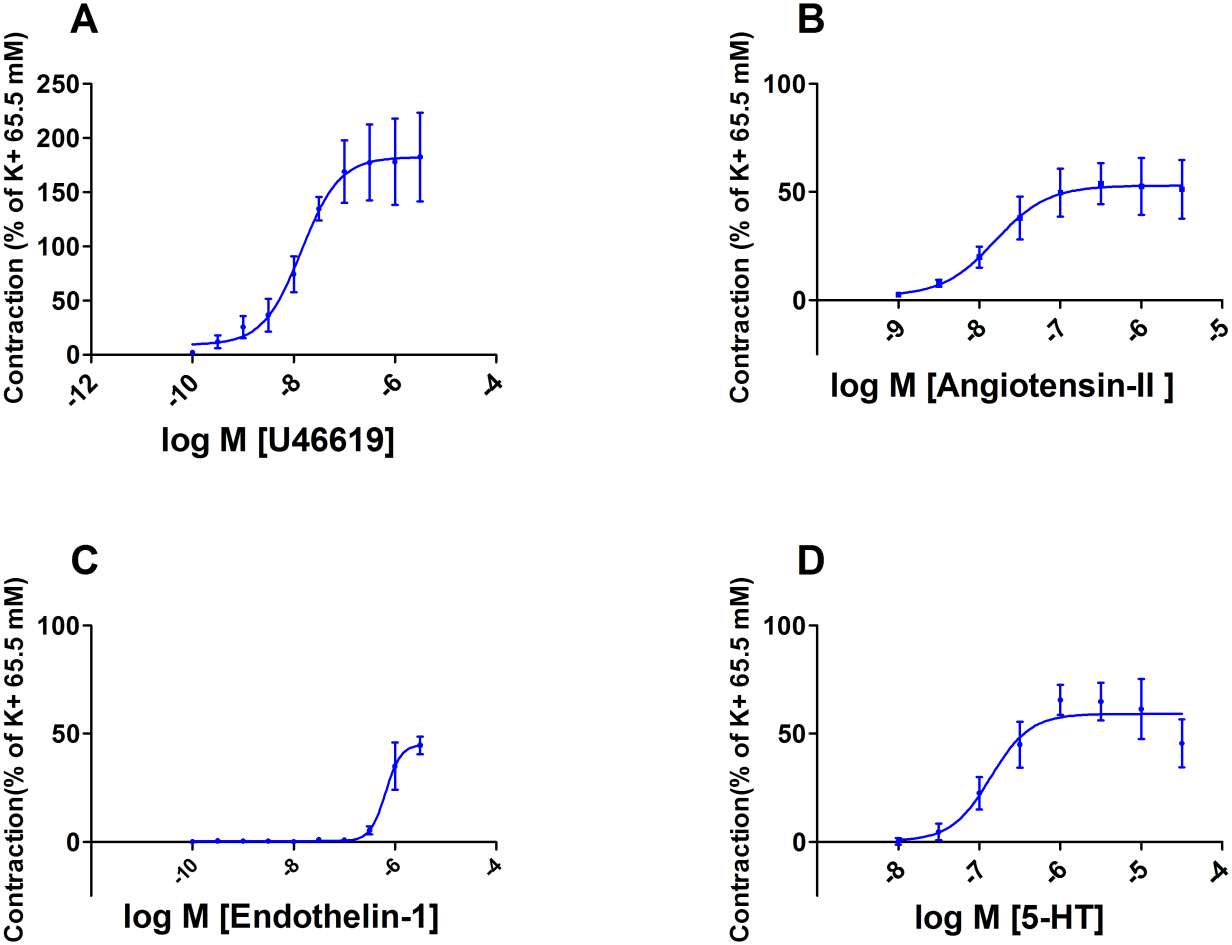
Vasoconstriction of chorionic arteries. Concentration-dependent contractions of placental chorionic arteries induced by U46619 (n = 3) (A), AngII (n = 3) (B), ET-1 (n = 4) (C), or 5-HT (n = 6) (D), compared to a pre-contraction induced by KCl 65.5 mM.

Dose-dependent vasoconstriction of chorionic arteries was tested for U46619, AngII, ET-1, 5-HT and BK. The maximal response related to the 65.5 mM KCl induced contraction was: 183 ± 71, 54 ± 17, 45 ± 8 and 66 ± 20% respectively. BK (1 nM– 3 μM, n = 3), had no effect on baseline wall-tension. A pD_2_ could be calculated from the concentration-dependent relaxation curves for U46619 (7.9 ± 0.4), AngII (7.8 ± 0.7), ET (6.2 ± 0.9), 5-HT (6.9 ± 0.4) (Fig 3).

### Tissue viability

LDH levels of the conditioned medium of both the standard culture (21% O_2_) and the severe hypoxic condition were compared. No significant difference (p = 0.1000) was found for LDH levels in the conditioned medium between the two conditions. This indicates that tissue viability was comparable between the two conditions after the 3 hours PSM collection procedure.

### Vasoactive responses of chorionic arteries to PSMs

**Fig 4:**
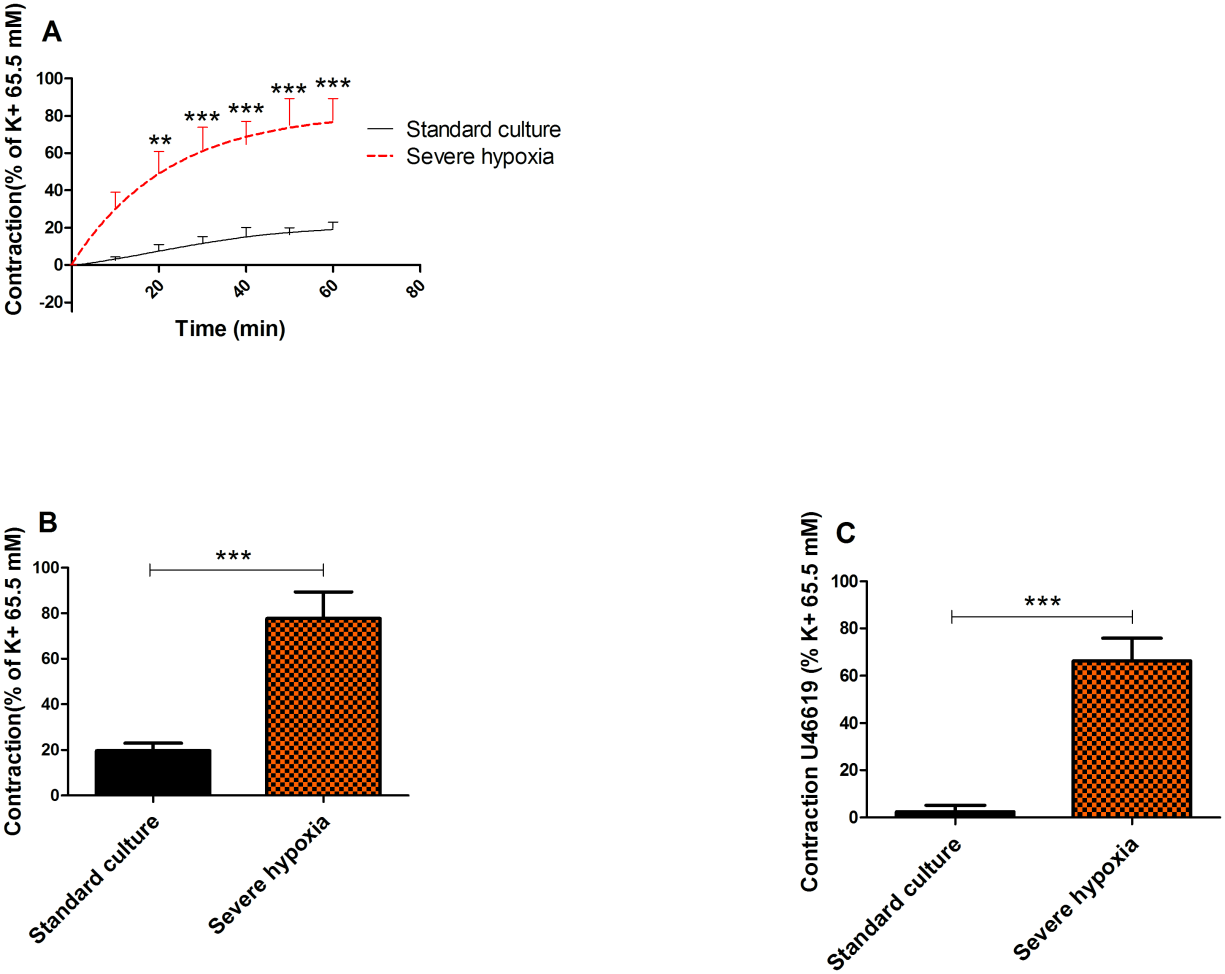
Vasoactive responses of chorionic arteries to PSMs. The vascular contractile response over time (60 min) induced by PSMs released during exposure to room air (control) (n = 8) or hypoxia (n = 8) (A). The maximum response after 60 min (n = 8) (B) and differences in vascular contractility to U46619 before and after exposure to PSMs for both conditions (n = 8) (C).

PSMs released under severe hypoxia resulted in a significant (p = 0.0002) time-dependent increased contraction of 78 ± 33% compared to the standard culture condition (20 ± 9%) (n = 8) after 1 hour (Fig 4A/4B). To test the sustained effects on vascular contractility to U46619, the contraction induced by U46619 (30 nM) before and after exposure to PSMs was determined (Fig 4C). PSMs released under severe hypoxic conditions induced an increased response to U46619 (64 ± 30%) compared to those produced by standard culture (21% O_2_) conditions (2 ± 8%; p = 0.0002) (Fig 4).

### Characterisation of vasoactive PSMs released under hypoxia

**Fig 5:**
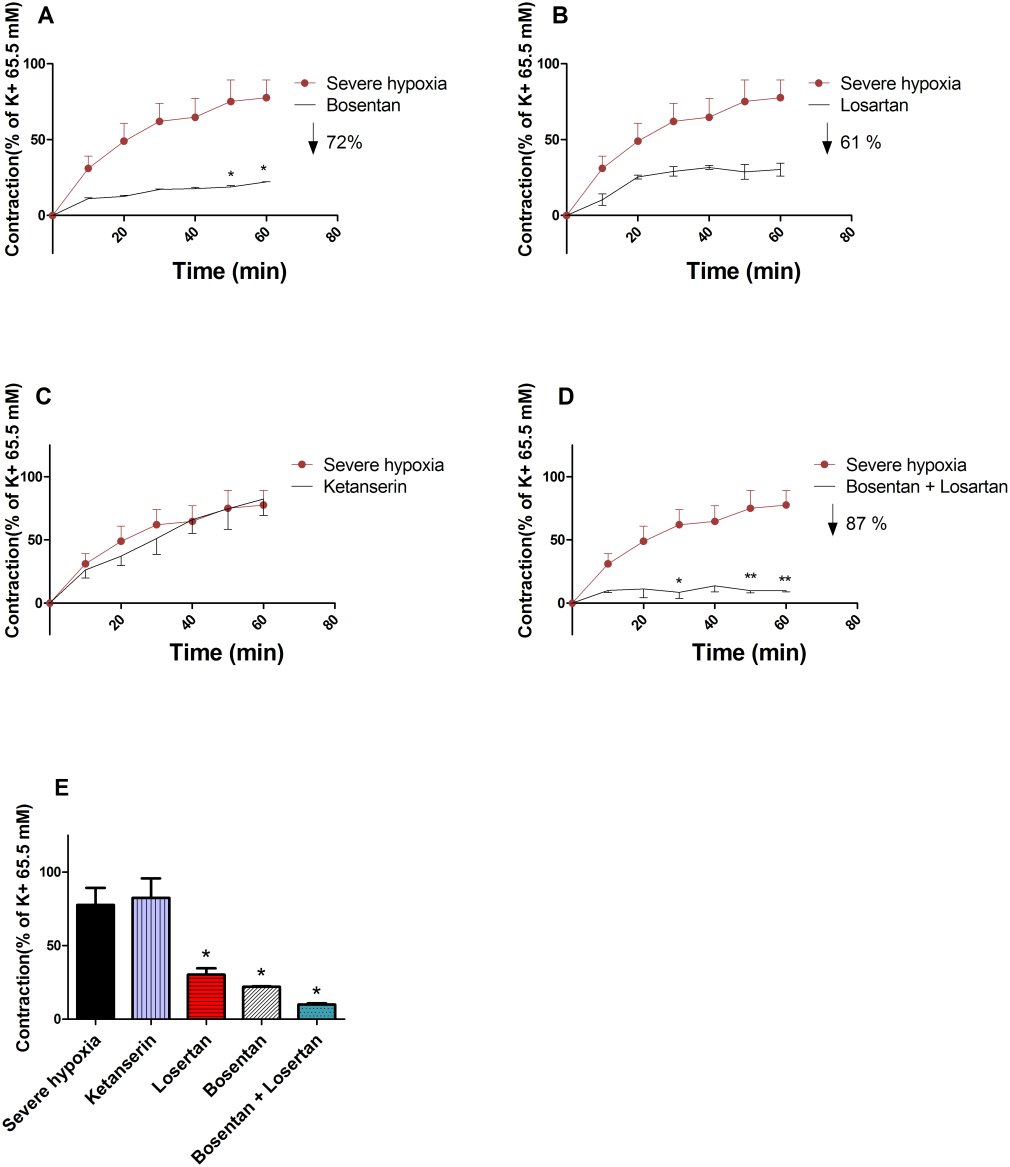
Characterization of vasoactive PSMs released under hypoxia. The vascular contractile response over time (60 min) induced by PSMs released under hypoxia in combination with a 15 min pre-incubation with either 30 μM Bos (n = 3) (A), or 30 μM Ket (n = 3) (B), or 30 μM Los (n = 3) (C), or 30 μM Bos + Los (n = 3) (D). The maximum response to PSMs released under hypoxia after 60 min (n = 3) (E).

PSMs released under severe hypoxia were added to chorionic arteries for 1 hour after a 15 min pre-incubation with the following antagonists: Bos, Los, Ket or Bos + Los. A 72 ± 1% decrease (p = 0.0121) in vascular contractile response to PSMs was evoked when vessels were pre-incubated with Bos (30 μM), a competitive ET-1 (A/B) receptor antagonist (Fig 5A and 5E). Los (30 μM), a competitive AT-1 receptor antagonist decreased (p = 0.0242) the PSMs induced contraction by 61 ± 8% (Fig 5B and 5E). The combination losartan and bosentan (30 μM) showed a decrease (p = 0.0121) of 87 ± 2% (Fig 5D and 5E). The contractile response was not affected (p = 0.9212) by Ket (30 μM), a competitive serotonin (5-HT_2_) antagonist (Fig 5C and 5E).

### Vascular contractile effects of ET-1 and AngII in isolation

AngII (3 μM), ET-I (3 μM) or the combination, induced vasoconstriction of 53 ± 19, 45 ± 8 and 112 ± 31% respectively, related to the contractile response of 65.5 mM KCl. The combination of ET-1 and AngII induced more vasoconstriction compared to the components in isolation, indicating that the pathways activated, are partially independent (n = 4) (data not shown).

### Consistency of the extraction procedure for PSMs

To test the reproducibility of our experimental setting the coefficient of the variance within one and between three extraction procedures was determined. PSMs released under severe hypoxia from one placenta, induced a similar vascular contraction in chorionic arteries isolated from 3 different placentas (CV = 4.5%). PSMs released under severe hypoxia from 3 different placentas also resulted in a similar (CV = 0.5%) vascular contraction in chorionic arteries isolated from three different placentas.

### Effects of quercetin on the release of PSMs under hypoxia

**Fig 6:**
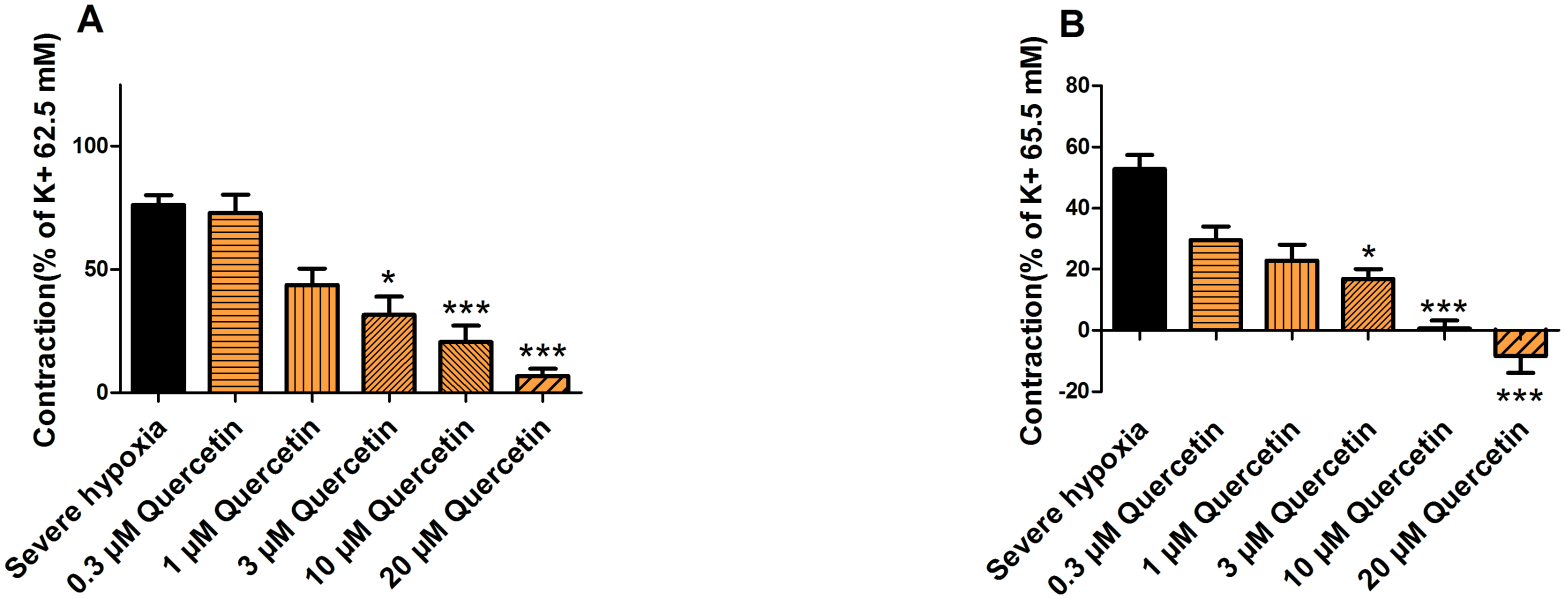
Effects of quercetin on the release of PSMs under hypoxia. The maximum vascular contractile response after 60 min induced by PSMs released under hypoxia (control) (n = 27), hypoxia in combination with 0.3, 1, 3, 10 or 20 μM quercetin (n = 8) (A) and the difference in vascular contractility to U46619 before and after exposure to the PSMs for each condition (B).

Quercetin (0.3, 1, 3, 10 or 20 μM) added to 200 ml pre-warmed HEPES buffer (37°C) containing 30 g placental tissue followed by 3 hours 100% N_2_ aeration resulted in a concentration-dependent decreased vasoconstriction of 4 ± 28, 43 ± 25, 58 ± 29, 73 ± 25 and 91 ± 12% respectively induced by the released PSMs (Fig 6A). Furthermore, a concentration-dependent decreased reactivity to U46619 of 23 ± 12, 30 ± 15, 36 ± 9, 52 ± 7 and 61 ± 17% was found (Fig 6B).

## Discussion

In this study, an *in vitro* model is presented for PE using placental villous explants of non-complicated term placentas and chorionic arteries. This model can be used to study the production of PSMs by the placenta that induce the acute vasoconstriction and sustained increased contractility observed in PE.

The vascular reactivity of chorionic arteries was characterised using various vasoactive compounds. Car was used to confirm that the absence of ACh-induced vasorelaxation was not due to the breakdown of ACh by cholinesterase. It was found that BK only induces vasorelaxation after a pre-contraction by U46619 (-40 ± 20%) and not after a pre-contraction by KCl, which is known to inhibit the release EDHF [27, 28]. It was therefore suggested that the vasorelaxation was induced by the released EDHF in response to BK [29]. Since BK did not induced vasoconstriction as previously observed in umbilical arteries, it was concluded that no TXA_2_ was produced in response to BK, which could have masked the BK-induced vasorelaxation [30, 31]. The endothelial independent vasorelaxator SNP induced a relaxation up to 76 ± 23% in KCl pre-contracted arteria and was thereby the most potent vasorelaxator. Even though ACh and BK did not induce a relevant NO-dependent vasorelaxation, SNP proved that the vessels used for our further experiments are NO-sensitive. Concentration-dependent vasoconstrictions were induced by U46619, AngII, ET-1, 5-HT with a maximal response related to the contractile response of 65.5 mM KCl of 183 ± 71, 54 ± 17, 45 ± 8 and 66 ± 20% respectively. This shows that arteries contained functional active AT-1, ET-1 and 5-HT_2_ receptors.

In our *in vitro* model, we found that PSMs released under severe hypoxia induced a 58% increase in vascular contraction compared to the PSMs obtained from the standard culture (21% O_2_) condition. Beside altered vasoconstriction, the PSMs released under severe hypoxia also caused a 64% sustained increase in vascular contractility to U46619. With the use of specific receptor antagonists, the AT-1 and ET-1 receptors were identified as the main contributors to the direct vasoconstrictive activity of the PSMs. These results obtained in our model are in line with the paradigm that stress factors, like hypoxia, in placental tissue lead to the release of PSMs causing acute high blood pressure and increased risk for high blood pressure in later life of the mother [5].

Khalil et al. already suggested that bioactive factors like AT-1-agonistic autoantibodies released by the placenta could cause severe vasoconstriction. Previously it has been proposed that besides activating the AT-1 receptor, increased levels of placental AT-1-agonistic autoantibodies may stimulate endothelial cells to produce ET-1, which may further trigger the genesis of preeclamptic hypertension [32].

The increased contractility of arteries after 1 hour pre-incubation with PSMs corresponds to a similar increased vascular contractility to TXA_2_, AngII and ET-1 of omental arteries observed in women suffering from PE [33–35]. Therefore, it will be one of the goals of our model to test the vascular effect of PSMs on omental arteries, which will further unravel the complex pathophysiological process of PE.

Although the exact mechanism of PE is still enigmatic, there is a growing body of evidence that vasoactive PSMs obtained under hypoxia promote a TXA_2_ and prostaglandins disbalance and that this is pivotal in the development of PE-induced hypertension [35]. It can explain the increased maternal vascular contractility to vasoactive compounds after a PE-complicated pregnancy [36]. Our data indicate that hypoxia is one of the triggers that lead to the release of vasoactive PSMs into the maternal circulation and forming the culprit of the acute increase in blood pressure during pregnancy as well as the increased risk for high blood pressure in later life of the mother.

The low inter- and intra-coefficient of the variance of the PSM extraction procedure of 4.5 and 0.5% respectively indicates the applicability of the PSM generation procedure under severe hypoxia and its induced vaso-reactivity in chorionic arteries.

Well-known key players in PE, like oxidative and inflammatory stress, are also known to be powerful stimuli for altered protein expression [37]. A better understanding of the changes in protein expression will allow the development of novel biomarkers for the prediction and diagnosis and potential treatment targeting key aspects of the pathophysiology [37]. The experiment with the dietary bioactive compound quercetin underlines the potential of our *in vitro* model. When quercetin, the most abundant flavonoid in our diet, was added to the HEPES buffer containing the placental tissue before exposure to severe hypoxia, the direct vasocontraction and increased vascular contractility to U46619 induced by the released PSMs was concentration-dependently reduced. Although the exact mode of action of quercetin needs to be elucidated, this indicates that bioactive compounds in our diet might be useful in targeting the different elements of PE. Moreover, antioxidants are known for their anti-inflammatory effect. Several molecular mechanisms are involved: e.g. via inhibition of the pro-inflammatory transcription factor nuclear factor-kappa B (NF-kB). Our lab recently established that many flavonoids inhibit Poly(ADP-ribose)polymerase-1 (PARP-1), which is known to have a coactivating function in the Nf-kB-mediated gene expression [38].

Further experiments can include exposure of placental tissue to inflammatory stress factors like TNF-α (Tumor necrosis factor-alpha) to determine whether only hypoxia triggers the release of vasoactive compounds by syncytiotrophoblasts cells. Previous studies showed evidence that HIF-1α (Hypoxia-inducible factor 1-alpha), considered as the master transcriptional regulator of the cellular and developmental response to hypoxia itself can be stimulated and stabilized by inflammatory stimuli like lipopolysaccharide (LPS) under normoxic conditions. This shows a potential overlap between inflammatory and hypoxic responses in preeclampsia [16, 39, 40]. Besides changes in vascular reaction, specific inflammatory cytokines and vesicles characteristics could be determined in the conditioned medium. Redman et al. speculated, that in PE due to oxidative and inflammatory stress, syncytiotrophoblast cells are activated to release larger microvesicles that exhibit proteins, which have pro-inflammatory, anti-angiogenic and procoagulant activity that could contribute to exaggerated systemic inflammation in PE [41]. It is now recognized that the exact nature and composition of syncytiotropoblast microvesicles has yet to be defined. However, *ex vivo* studies have shown that these microvesicles play a key role in normal maternal immunomodulation that results in the regulation of the T-helper cells and may be thus responsible for the Th1 skewness in PE [42, 43]. As described by Wareing et al. the use of a humidified gas mixture of 1, 6 or 20% O_2_ with 5% CO_2_ and N_2_ in our model would ameliorate the relevance of mimicking physiological and pathophysiological intervillous oxygen tensions [44].

In conclusion, an *in vitro* model for PE was set up using healthy term placentas and chorionic arteries to investigate the direct and sustained vascular consequences of PSMs released under hypoxic stress conditions in the placenta as observed in PE. The model is also suitable to test the potential beneficial effects of food compounds like quercetin to prevent the vascular consequences of PE and to elucidate their mechanism of action. Our *in vitro* model opens avenues to develop interventions aimed at treating or even preventing preterm birth in PE.

## Abbreviations

5-HT: Serotonin
ACh: Acetylcholine
AT-1: Angiotensin-II type 1 receptor
AngII: Angiotensin-II
BK: Bradykinin
Bos: Bosentan
Car: Carbachol
CRH: Corticotrophin-releasing hormone
EDHF: Endothelium-derived hyperpolarizing factor
ET-1: Endothelin-1
EVTs: Extra villous trophoblast
HIF-1α: Hypoxia-inducible factor 1-alpha
HLA: Human leukocyte antigen
Ket: Ketanserin
KIR: Immunoglobulin-like receptors
LDH: Lactate dehydrogenase
Los: Losartan
NF-kB: nuclear factor-kappa B
PARP: Poly(ADP-ribose)polymerase-1
PE: Preeclampsia
PSMs: Placental secreted messengers
ROS: Reactive oxygen species
SNP: Sodium nitroprusside
TNF-α: Tumor necrosis factor-alpha
TXA_2_: Thromboxane A2
VSM: Vasculosyncytial membrane

## References

[1] Saleh L, Verdonk K, Visser W, van den Meiracker AH, Danser AH. The emerging role of endothelin-1 in the pathogenesis of pre-eclampsia. Ther Adv Cardiovasc Dis. 2016 10.1177/1753944715624853 . 26755746 PMC5933567

[2] Drenthen W, Boersma E, Balci A, Moons P, Roos-Hesselink JW, Mulder BJ, et al Predictors of pregnancy complications in women with congenital heart disease. Eur Heart J. 2010;31(17):2124–32. 10.1093/eurheartj/ehq200 . 20584777

[3] Steegers EA, von Dadelszen P, Duvekot JJ, Pijnenborg R. Pre-eclampsia. Lancet. 2010;376(9741):631–44. 10.1016/S0140-6736(10)60279-6 . 20598363

[4] Parets SE, Bedient CE, Menon R, Smith AK. Preterm birth and its long-term effects: methylation to mechanisms. Biology (Basel). 2014;3(3):498–513. 10.3390/biology3030498 . 25256426 PMC4192624

[5] Morton JS, Cooke CL, Davidge ST. In Utero Origins of Hypertension: Mechanisms and Targets for Therapy. Physiol Rev. 2016;96(2):549–603. 10.1152/physrev.00015.2015 . 26887677

[6] Nakimuli A, Chazara O, Farrell L, Hiby SE, Tukwasibwe S, Knee O, et al Killer cell immunoglobulin-like receptor (KIR) genes and their HLA-C ligands in a Ugandan population. Immunogenetics. 2013;65(11):765–75. 10.1007/s00251-013-0724-7 . 23974321 PMC3824577

[7] Moffett A, Hiby SE. How Does the maternal immune system contribute to the development of pre-eclampsia? Placenta. 2007;28 Suppl A:S51–6. 10.1016/j.placenta.2006.11.008 . 17292469

[8] Burton GJ, Woods AW, Jauniaux E, Kingdom JC. Rheological and physiological consequences of conversion of the maternal spiral arteries for uteroplacental blood flow during human pregnancy. Placenta. 2009;30(6):473–82. 10.1016/j.placenta.2009.02.009 . 19375795 PMC2697319

[9] Kaufmann P, Black S, Huppertz B. Endovascular trophoblast invasion: implications for the pathogenesis of intrauterine growth retardation and preeclampsia. Biol Reprod. 2003;69(1):1–7. 10.1095/biolreprod.102.014977 . 12620937

[10] Roberts JM, Escudero C. The placenta in preeclampsia. Pregnancy Hypertens. 2012;2(2):72–83. 10.1016/j.preghy.2012.01.001 . 22745921 PMC3381433

[11] Crocker I. Gabor Than Award Lecture 2006: pre-eclampsia and villous trophoblast turnover: perspectives and possibilities. Placenta. 2007;28:S4–S13. 10.1016/j.placenta.2007.01.016 17379302

[12] Heazell AE, Taylor NN, Greenwood SL, Baker PN, Crocker IP. Does altered oxygenation or reactive oxygen species alter cell turnover of BeWo choriocarcinoma cells? Reprod Biomed Online. 2009;18(1):111–9. . 19146777 10.1016/s1472-6483(10)60432-4

[13] Wu F, Tian FJ, Lin Y. Oxidative Stress in Placenta: Health and Diseases. Biomed Res Int. 2015;2015:293271 10.1155/2015/293271 . 26693479 PMC4676991

[14] Myatt L, Cui X. Oxidative stress in the placenta. Histochem Cell Biol. 2004;122(4):369–82. 10.1007/s00418-004-0677-x . 15248072

[15] Huppertz B. Placental Villous Trophoblast: the Altered Balance Between Proliferation and Apoptosis Triggers Pre-eclampsia Journal of Reproductive Medicine and Endocrinology 2006;J. Reproduktionsmed. Endokrinol 2006; 3 (2), 103–108.

[16] Redman CW, Sargent IL. Placental stress and pre-eclampsia: a revised view. Placenta. 2009;30 Suppl A:S38–42. 10.1016/j.placenta.2008.11.021 . 19138798

[17] Goulopoulou S, Davidge ST. Molecular mechanisms of maternal vascular dysfunction in preeclampsia. Trends Mol Med. 2015;21(2):88–97. 10.1016/j.molmed.2014.11.009 . 25541377

[18] Redman CW, Sargent IL. Preeclampsia and the systemic inflammatory response. Semin Nephrol. 2004;24(6):565–70. . 15529291 10.1016/s0270-9295(04)00127-5

[19] Luft FC. Proinflammatory effects of angiotensin II and endothelin: targets for progression of cardiovascular and renal diseases. Curr Opin Nephrol Hypertens. 2002;11(1):59–66. . 11753088 10.1097/00041552-200201000-00009

[20] Newson AJ. Ethical aspects arising from non-invasive fetal diagnosis. Semin Fetal Neonatal Med. 2008;13(2):103–8. 10.1016/j.siny.2007.12.004 . 18243828

[21] Blehar MC, Spong C, Grady C, Goldkind SF, Sahin L, Clayton JA. Enrolling pregnant women: issues in clinical research. Womens Health Issues. 2013;23(1):e39–45. 10.1016/j.whi.2012.10.003 . 23312713 PMC3547525

[22] Boots AW, Haenen GR, Bast A. Health effects of quercetin: from antioxidant to nutraceutical. Eur J Pharmacol. 2008;585(2–3):325–37. 10.1016/j.ejphar.2008.03.008 . 18417116

[23] Hytten F. Blood volume changes in normal pregnancy. Clin Haematol. 1985;14(3):601–12. . 4075604

[24] Perry IJ, Beevers DG, Whincup PH, Bareford D. Predictors of ratio of placental weight to fetal weight in multiethnic community. BMJ. 1995;310(6977):436–9. . 7873949 10.1136/bmj.310.6977.436 PMC2548818

[25] Butler IB, Schoonen MA, Rickard DT. Removal of dissolved oxygen from water: a comparison of four common techniques. Talanta. 1994;41(2):211–5. 18965910 10.1016/0039-9140(94)80110-x

[26] Szatanek R, Baran J, Siedlar M, Baj-Krzyworzeka M. Isolation of extracellular vesicles: Determining the correct approach (Review). Int J Mol Med. 2015;36(1):11–7. 10.3892/ijmm.2015.2194 . 25902369 PMC4494580

[27] Gillham JC, Kenny LC, Baker PN. An overview of endothelium-derived hyperpolarising factor (EDHF) in normal and compromised pregnancies. Eur J Obstet Gynecol Reprod Biol. 2003;109(1):2–7. . 12818435 10.1016/s0301-2115(03)00044-7

[28] Lemkens P, Spijkers LJ, Meens MJ, Nelissen J, Janssen B, Peters SL, et al Dual NEP/ECE inhibition improves endothelial function in mesenteric resistance arteries of 32-week-old SHR. Hypertens Res. 2017;40(8):738–45. 10.1038/hr.2017.38 . 28298655

[29] Nishikawa Y, Stepp DW, Chilian WM. Nitric oxide exerts feedback inhibition on EDHF-induced coronary arteriolar dilation in vivo. Am J Physiol Heart Circ Physiol. 2000;279(2):H459–65. 10.1152/ajpheart.2000.279.2.H459 . 10924042

[30] Wilkes BM, Hollander AM, Sung SY, Mento PF. Cyclooxygenase inhibitors blunt thromboxane action in human placental arteries by blocking thromboxane receptors. Am J Physiol. 1992;263(4 Pt 1):E718–23. 10.1152/ajpendo.1992.263.4.E718 . 1415690

[31] Zhao S Wa Y. Vascular Biology of the Placenta, Chapter 8: Vasoactivators and Placental Vasoactivity 2010.

[32] George EM, Granger JP. Endothelin: key mediator of hypertension in preeclampsia. Am J Hypertens. 2011;24(9):964–9. 10.1038/ajh.2011.99 . 21677700 PMC3388717

[33] Belfort MA, Saade GR, Suresh M, Kramer W, Vedernikov YP. Effects of selected vasoconstrictor agonists on isolated omental artery from premenopausal nonpregnant women and from normal and preeclamptic pregnant women. Am J Obstet Gynecol. 1996;174(2):687–93. . 8623808 10.1016/s0002-9378(96)70451-9

[34] Everett RB, Worley RJ, MacDonald PC, Gant NF. Effect of prostaglandin synthetase inhibitors on pressor response to angiotensin II in human pregnancy. J Clin Endocrinol Metab. 1978;46(6):1007–10. 10.1210/jcem-46-6-1007 . 122438

[35] Walsh S, Glob. libr. women’s med., (ISSN: 1756-2228) 2011.

[36] Walsh SW. Preeclampsia: an imbalance in placental prostacyclin and thromboxane production. Am J Obstet Gynecol. 1985;152(3):335–40. . 3923838 10.1016/s0002-9378(85)80223-4

[37] Tannetta D, Sargent I. Placental disease and the maternal syndrome of preeclampsia: missing links? Curr Hypertens Rep. 2013;15(6):590–9. 10.1007/s11906-013-0395-7 24108542 PMC3838579

[38] Geraets L, Moonen HJ, Brauers K, Wouters EF, Bast A, Hageman GJ. Dietary flavones and flavonoles are inhibitors of poly(ADP-ribose)polymerase-1 in pulmonary epithelial cells. J Nutr. 2007;137(10):2190–5. Epub 2007/09/22. 10.1093/jn/137.10.2190 . 17884996

[39] Blouin CC, Page EL, Soucy GM, Richard DE. Hypoxic gene activation by lipopolysaccharide in macrophages: implication of hypoxia-inducible factor 1alpha. Blood. 2004;103(3):1124–30. 10.1182/blood-2003-07-2427 . 14525767

[40] Possomato-Vieira JS, Khalil RA. Mechanisms of Endothelial Dysfunction in Hypertensive Pregnancy and Preeclampsia. Adv Pharmacol. 2016;77:361–431. 10.1016/bs.apha.2016.04.008 . 27451103 PMC4965238

[41] Redman CW, Tannetta DS, Dragovic RA, Gardiner C, Southcombe JH, Collett GP, et al Review: Does size matter? Placental debris and the pathophysiology of pre-eclampsia. Placenta. 2012;33 Suppl:S48–54. 10.1016/j.placenta.2011.12.006 . 22217911

[42] Tannetta D, Masliukaite I, Vatish M, Redman C, Sargent I. Update of syncytiotrophoblast derived extracellular vesicles in normal pregnancy and preeclampsia. J Reprod Immunol. 2017;119:98–106. 10.1016/j.jri.2016.08.008 . 27613663

[43] Pillay P, Moodley K, Moodley J, Mackraj I. Placenta-derived exosomes: potential biomarkers of preeclampsia. Int J Nanomedicine. 2017;12:8009–23. 10.2147/IJN.S142732 . 29184401 PMC5673050

[44] Robinson NJ, Wareing M, Hudson NK, Blankley RT, Baker PN, Aplin JD, et al Oxygen and the liberation of placental factors responsible for vascular compromise. Lab Invest. 2008;88(3):293–305. Epub 2008/01/30. 10.1038/labinvest.3700746 . 18227808

